# Antibiotic Resistance Genes, Virulence Factors, and Biofilm Formation in Coagulase-Negative *Staphylococcus* spp. Isolates from European Hakes (*Merluccius merluccius,* L.) Caught in the Northeast Atlantic Ocean

**DOI:** 10.3390/pathogens12121447

**Published:** 2023-12-13

**Authors:** Lara Díaz-Formoso, Vanessa Silva, Diogo Contente, Javier Feito, Pablo E. Hernández, Juan Borrero, Gilberto Igrejas, Rosa del Campo, Estefanía Muñoz-Atienza, Patrícia Poeta, Luis M. Cintas

**Affiliations:** 1 Grupo de Seguridad y Calidad de los Alimentos por Bacterias Lácticas, Bacteriocinas y Probióticos (Grupo SEGABALBP), Sección Departamental de Nutrición y Ciencia de los Alimentos (Nutrición, Bromatología, Higiene y Seguridad Alimentaria), Facultad de Veterinaria, Universidad Complutense de Madrid, Avda. Puerta de Hierro, s/n, 28040 Madrid, Spain; lardia01@ucm.es (L.D.-F.); diogodas@ucm.es (D.C.); ehernan@vet.ucm.es (P.E.H.); jborrero@ucm.es (J.B.); lcintas@vet.ucm.es (L.M.C.); 2 Microbiology and Antibiotic Resistance Team (MicroART), Department of Veterinary Sciences, University of Trás-os-Montes and Alto Douro (UTAD), 5000-801 Vila Real, Portugal; vanessasilva@utad.pt (V.S.); ppoeta@utad.pt (P.P.); 3 Department of Genetics and Biotechnology, University of Trás-os-Montes and Alto Douro (UTAD), 5000-801 Vila Real, Portugal; gigrejas@utad.pt; 4 Functional Genomics and Proteomics Unit, University of Trás-os-Montes and Alto Douro (UTAD), 5000-801 Vila Real, Portugal; 5 LAQV-REQUIMTE, Department of Chemistry, NOVA School of Science and Technology, Universidade Nova de Lisboa, 2829-516 Caparica, Portugal; 6 Servicio de Microbiología, Hospital Universitario Ramón y Cajal, Instituto Ramón y Cajal de Investigación Sanitaria (IRYCIS), 28034 Madrid, Spain; rosacampo@yahoo.com; 7 CECAV—Veterinary and Animal Research Centre, University of Trás-os-Montes and Alto Douro (UTAD), 5000-801 Vila Real, Portugal; 8 Associate Laboratory for Animal and Veterinary Science (AL4AnimalS), University of Trás-os-Montes and Alto Douro (UTAD), 5000-801 Vila Real, Portugal

**Keywords:** European hakes (*Merluccius merluccius*, L.), *Staphylococcus* spp., antimicrobial activity, antibiotic resistance, virulence factors, biofilm formation

## Abstract

The indiscriminate use of antibiotics has contributed to the dissemination of multiresistant bacteria, which represents a public health concern. The aim of this work was to characterize 27 coagulase-negative staphylococci (CoNS) isolated from eight wild Northeast Atlantic hakes (*Merluccius merluccius*, L.) and taxonomically identified as *Staphylococcus epidermidis* (*n* = 16), *Staphylococcus saprophyticus* (*n* = 4), *Staphylococcus hominis* (*n* = 3), *Staphylococcus pasteuri* (*n* = 2), *Staphylococcus edaphicus* (*n* = 1), and *Staphylococcus capitis* (*n* = 1). Biofilm formation was evaluated with a microtiter assay, antibiotic susceptibility testing was performed using the disk diffusion method, and antibiotic resistance and virulence determinants were detected by PCR. Our results showed that all staphylococci produced biofilms and that 92.6% of the isolates were resistant to at least one antibiotic, mainly penicillin (88.8%), fusidic acid (40.7%), and erythromycin (37%). The penicillin resistance gene (*blaZ*) was detected in 66.6% (18) of the isolates, of which 10 also carried resistance genes to macrolides and lincosamides (*mphC*, *msr(A/B)*, *lnuA*, or *vgaA*), 4 to fusidic acid (*fusB*), and 3 to trimethoprim-sulfamethoxazole (*dfrA*). At least one virulence gene (*scn*, *hla*, *SCCmecIII*, and/or *SCCmecV*) was detected in 48% of the isolates. This study suggests that wild European hake destined for human consumption could act as a vector of CoNS carrying antibiotic resistance genes and/or virulence factors.

## 1. Introduction

Marine fisheries are part of the primary extractive sector, comprising the maritime areas defined by the Food and Agriculture Organization (FAO, Rome, Italy) of the United Nations (New York, NY, USA), with precise delimitations for each major fishing area [1]. The importance of responsible use of fishery and aquaculture resources is now recognized. In 2020, the yield from capture fisheries amounted to 90.3 million tonnes [2]. Fish and fishery products continue to be amongst the most traded food products worldwide due to the fact that fish are a significant source of protein and minerals. The global consumption of edible fish increased at an average annual rate of 5.9% between 1960 and 2020. For 3.3 billion people, aquatic foods represent at least 20% of the average per capita intake of animal protein [2]. In the Spanish gastronomic culture, European hake (*Merluccius merluccius*, L.) is one of the most deeply rooted and valued white fish, with an important economic impact on the fishing sector. This fish species is mostly derived from the catches of the Spanish fishing fleet and from intra-Community trade [3]. Specifically, 62,500 tonnes of European hake were consumed in Spain in 2021 [4].

Traditionally, antibiotics have been used as therapeutic agents and, in some cases, as prophylactic treatment of bacterial ichthyopathologies. In this respect, the overuse of antibiotics has contributed to the growing and serious problem of the emergence and spread of transmissible bacterial resistance genes to many antibiotics, which is a major global problem for the treatment of infectious diseases of bacterial etiology [5], endangering veterinary and human medicine and affecting food safety and the environment [1]. The most commonly used antibiotics in aquaculture are amoxicillin, florfenicol, oxytetracycline, oxolinic acid, flumequine, enrofloxacin, and trimethoprim-sulfadiazine [6,7,8].

In the European Union (EU), and in most industrialized countries, their use as a prophylactic treatment has been expressly prohibited, and increasingly restrictive regulations on their use have been developed due to their serious adverse effects on animal and human health, food safety, and the environment [9,10,11]. In 2006, the EU banned the use of antibiotics as growth promoters due to the increasing spread of antimicrobial-resistant bacteria [12]. Additionally, Commission Regulation (EU) 37/2010 sets maximum residue limits (MRLs) on pharmacologically active substances in foodstuffs of animal origin [13]. However, the use of antibiotics is not strictly regulated in all countries, making it difficult to control the spread of bacterial resistance [14]. In 2015, a total of 8361 tonnes of antimicrobial agents were used in veterinary practices in the EU [15] and, according to a 2017 ECDC/EFSA/EMA report, tetracycline and penicillin were the most prescribed antibiotics for food-producing animals [16]. Through the consumption of food of animal origin, including fish, humans can be exposed to antibiotic residues and bacteria carrying resistance genes [17]. In 2019, five million people died globally from causes related to bacterial antimicrobial resistance, and 1.3 million people died as a direct result of antibiotic-resistant bacteria [18]. If no action is taken by 2050, these diseases could cause 10 million deaths per year, representing the leading cause of death globally [18,19]. Considering the growing concern about the risk of antibiotic resistance and its possible worsening in the future, collaboration between health authorities has been encouraged to develop strategies focused on the correct use of veterinary medicines to prevent bacterial resistance, such as the current engagement between the FAO, the World Organization for Animal Health (OIE) and the World Health Organization (WHO) to address the challenge of antimicrobial resistance to human health worldwide [20].

Species of the genus *Staphylococcus* are recognized as pathogens responsible for several opportunistic diseases in humans and animals [21] and are the most frequent cause of biofilm-associated infections [22]. Although coagulase-negative staphylococci (CoNS) are not classical food poisoning bacteria, a number of studies indicate that food can be considered as an important route for the transmission of antibiotic-resistant CoNS harboring multiple antibiotic resistance genes [23]. Although some species of CoNS have been described as starter cultures playing a valuable role in the fermentation and biopreservation of meat products [24], other species such as *Staphylococcus epidermidis*, *Staphylococcus haemolyticus*, and *Staphylococcus saprophyticus* are considered as emerging opportunistic pathogens [25]. Particularly, *S. epidermidis* is the most clinically relevant and characterized biofilm-forming microorganism [26]. Staphylococcal bacteria isolated from different origins, including humans, livestock, companion animals, and food, have largely been characterized to determine the presence of antibiotic resistance genes [27,28,29,30,31]. The most frequent mechanisms of acquired resistance in staphylococci include the acquisition of *blaZ*, which encodes the production of a β-lactamase enzyme (conferring resistance to penicillin); *mecA*, which encodes the expression of PBP2 (conferring resistance to methicillin and cephalosporins); *tet*K and *tet*L, which encode the production of efflux pumps, and *tet*M or *tet*O, which encode the elongation factor-like proteins protecting ribosomes (conferring resistance to tetracycline); *aac(6′)-Ie-aph(2″)-Ia*, *aph(3′)-IIIa*, *ant(4′)-Ia*, and *str*, which encode cytoplasmic aminoglycoside-modifying enzymes; *erm*A, *erm*B, *erm*C, and *mph*C, which are responsible for target modification, and *msr*(A/B), *lnu*A, *lnu*B, and *vga*A, which are responsible for target protection (conferring resistance to macrolides and lincosamides); *fus*B, which confers target protection for fusidic acid resistance; and *dfr*A, *dfr*D, *dfr*G, and *dfr*K, which encode dihydrofolate reductase (DHFR) enzymes that are not susceptible to the inhibition of trimethoprim [32].

Recently, there has been great interest in elucidating whether the spread of antibiotic-resistant staphylococci from humans and livestock reaches the marine environment and, consequently, wild fish that may act as reservoirs [33,34,35]. For this reason, and taking into consideration that European hake is one of the most consumed fish in Spain and an unexplored niche, the aim of this work was to characterize staphylococcal isolates from intestinal samples of eight hake from the Northeast Atlantic to study their resistance to antibiotics, the presence of virulence factor genes, and their capability to produce biofilms.

## 2. Materials and Methods

### 2.1. Fish Collection

Eight European hake (*Merluccius merluccius*, L.) specimens (1.0–1.5 kg approx.), caught by a Galician professional fishing skipper in the Northeast Atlantic, specifically in the sub-area 27.VIIj (Southwest of Ireland) [36], during two consecutive years (June 2021 and 2022), were used for bacterial isolation.

### 2.2. Sample Collection, Bacterial Isolation and Antimicrobial Activity Assays

The fish were transported in polystyrene boxes with ice under refrigeration and aseptically handled until arrival at the laboratory within 48–72 h of their capture. One gram of feces of each hake was extracted and then diluted in peptone water (Oxoid Ltd., Basingstoke, UK) and pour-plated onto De Man, Rogosa, and Sharpe (MRS, Oxoid) agar (1.5%, *w*/*v*, Scharlau, Barcelona, Spain) plates. For the collection of intestinal samples, the gut was rinsed with 10 mM phosphate buffered saline (PBS, Oxoid Ltd., Basingstoke, UK), cut, and homogenized in a stomacher with peptone water. Subsequently, samples were pour-plated onto MRS agar plates. After plate incubation at 30 °C under aerobic and anaerobic conditions for 24–72 h, a total of 286 isolates with different morphologies were selected and tested for direct antimicrobial activity, using the Stab-On-Agar Test (SOAT) [37], against several ichthyopathogens of relevance to aquaculture (i.e., *Lactococcus garvieae* CF00021, *Lc. garvieae* CLG4, *Listeria monocytogenes* CECT911, *Listeria ivanovii* CECT913, *Yersinia ruckeri* LMG3279, *Aeromonas hydrophila* CECT839, *A. hydrophila* CECT5734, *A. hydrophila* CECT5734, *Aeromonas salmonicida* CLFP-23, *A. salmonicida* CECT4237, *Listonella anguillarum* CECT4344, *Tenacibaculum maritimum* NCIMB2154, *T. maritimum* CECT1161, *Edwardsiella tarda* CECT886, and *Streptococcus parauberis* LMG22225). A total of 66 isolates were preselected based on their direct antimicrobial activity.

### 2.3. Taxonomic Identification of Bacterial Isolates

Total bacterial DNA from the preselected 66 isolates was extracted by using the InstaGene Matrix (BioRad Laboratories, Inc., Hercules, CA, USA) according to the manufacturer’s instructions. The *16S rDNA* gene was amplified with the polymerase chain reaction (PCR) and then sequenced. PCR amplifications were performed using 25 µL of DreamTaq Hot Start PCR Master Mix 2x (Thermo Scientific, Waltham, MA, USA), 0.5 µM of fD1 (5′-AGAGAGTTTGATCCTGGCTCAG-3′), 0.5 µM rD1 (5′-TAAGGAGGAGGTGATCCAGCC-3′), 50–100 ng of purified DNA, and 19 µL of molecular biology–grade water (Thermo Scientific) [38]. PCR mixtures were subjected to several amplification cycles, starting with an initial denaturation cycle (95 °C, 3 min), followed by 35 cycles of denaturation (95 °C, 30 s), hybridization (60 °C, 30 s), and elongation (72 °C, 1 min) and a final elongation cycle (72 °C, 5 min) in a thermal cycler (Eppendorf, Hamburg, Germany). The resulting amplicons were then purified using the NucleoSpin^®^ Gel and PCR Clean-up kit (Macherey-Nagel™) and sent to Eurofins Genomics (Ebersberg, Germany) for DNA sequencing. To determine their taxonomic identification, the nucleotide sequences were analyzed using the BLAST nucleotide server of the National Center for Biotechnology Information (NCBI) (https://blast.ncbi.nlm.nih.gov/, accessed on 1 February 2023). The 27 CoNS isolates (Supplementary Table S1) were selected for further characterization.

### 2.4. Genetic Diversity Analysis by Enterobacterial Repetitive Intergenic Consensus—PCR (ERIC-PCR)

In order to study the genetic diversity of the CoNS isolated from European hakes, a PCR-based typing method was carried out. In particular, ERIC-PCR analysis of the 27 CoNS isolates was performed using primers ERIC-1R (5′-ATGTAAGCTCCTGGGGGGATTCAC-3′) and ERIC-2 (5′-AAGTAAGTGACTGGGGGGTGAGCG-3′) as previously described by Araújo et al. (2015) [39]. PCRs of 50 μL were prepared with 25 μL of MyTaq Mix (Bioline Reagents, Ltd., London, UK), 0.7 µM of each primer, 50–100 ng of purified DNA, 3 μM of MgCl_2_, and 19 µL of molecular biology–grade water. PCR mixtures were subjected to an initial denaturation (95 °C, 1 min), 35 cycles of denaturation-annealing-elongation (95 °C, 15 s; 46 °C, 15 s; and 72 °C, 10 s), and a final elongation (72 °C, 4 min) in a thermal cycler (Eppendorf, Hamburg, Germany). The amplification products were run at 90 V for 60 min in an electrophoresis chamber (BioRad Laboratories, Inc.), and the visualization of the bands was performed using the ChemiDoc Imaging System (BioRad Laboratories, Inc.), with HyperLadder 100 bp (Bioline Reagents, Ltd.) as a molecular weight marker. ERIC type analysis, clustering, and dendrogram construction were performed using Phoretix v.5.0 software (Nonlinear Dynamics Ltd., Newcastle upon Tyne, UK).

### 2.5. Biofilm Formation and Quantification Assays

Biofilm formation was tested using a microtiter assay as previously described by Oniciuc et al. (2016) [40]. The CoNS isolates were grown on tryptic soy agar (TSA, Oxoid) plates at 37 °C for 24 h. After incubation, two colonies were transferred to 3 mL tubes of tryptic soy broth (TSB, Oxoid) and incubated at 37°C with continuous shaking at 120 rpm (ES-80 Shaker-incubator, Grant Instruments, Cambridge, UK) for 16 h. Then, 200 µL of each bacterial suspension with a concentration of 1 × 10^6^ cfu/mL was added to each well of a 96-well flat-bottom microtiter plate (Orange Scientific, Braine-l’Alleud, Belgium). In all microplates, *Staphylococcus aureus* ATCC25923 and TSB without bacterial inoculum were used as the positive and negative controls, respectively. The microplates were incubated at 37 °C under aerobic conditions for 24 h. All experiments were carried out in sixteen replicates. Biofilm formation was quantified using the crystal violet (CV) staining method as previously described by Peeters et al. (2008) with some modifications [41]. Briefly, after microplate incubation, the medium was removed from each well by washing the plates twice with distilled water in order to remove unattached bacterial cells. The microplates were air-dried at room temperature for 30 min. To fix the biofilms, 100 µL of methanol (VWR International) was added to each well. After 15 min, the methanol was removed, the microplates were air-dried at room temperature for 10 min, and 100 µL of CV (1%, *v/v*) was added to each well. After 10 min, the CV was removed, and the microplates were washed twice with distilled water to remove the excess of dye and dried with a paper. Then, to solubilize the CV, 100 µL of acetic acid (33%, *v/v*) was added, and the absorbance was measured at 570 nm using a BioTek ELx808U microplate reader (BioTek, Winooski, VT, USA). To standardize the results, the biofilm of each isolate was normalized according to the results obtained with the positive control strain, *S. aureus* ATCC25923, assuming that it possessed a 100% biofilm-forming capability.

### 2.6. Antibiotic Susceptibility Testing

Antibiotic susceptibility testing was performed with the agar disk diffusion test according to the European Committee on Antimicrobial Susceptibility Testing (EUCAST) guidelines [42], except for kanamycin, for which the recommendations of the Clinical and Laboratory Standards Institute (CLSI) were followed [43]. For this purpose, the isolates were grown on TSA plates (Oxoid) at 37 °C for 24 h. Afterward, a colony was transferred to 3 mL tubes of sterile saline solution (0.9%, *w/v*) and seeded on Mueller Hinton agar (Oxoid). Disks containing known amounts of each antibiotic were placed on the surface of the agar plates, which were then incubated at 37 °C for 16 h. For this test, a total of 14 antibiotics were used: cefoxitin (30 μg), chloramphenicol (30 μg), ciprofloxacin (5 μg), clindamycin (2 μg), erythromycin (15 μg), fusidic acid (10 μg), gentamycin (10 μg), kanamycin (30 μg), linezolid (10 μg), mupirocin (200 μg), penicillin (1 U), tetracycline (30 μg), tobramycin (10 μg), and trimethoprim-sulfamethoxazole (1.25/23.75 μg). The susceptibility of CoNS to vancomycin was determined using a microdilution method in order to determine the minimum inhibitory concentration (MIC) following the guidelines provided by EUCAST (2023) [42]. Briefly, individual colonies were suspended in a sterile glass tube containing 10 mL saline solution (0.85% NaCl) to a turbidity of 0.5 on the McFarland scale, and then, the bacterial suspensions were diluted 1000-fold in Mueller Hinton broth (Oxoid). A volume of 50 μL of the diluted bacterial suspensions was added to each microplate well containing 50 μL of Mueller Hinton broth with vancomycin (1–64 µg/mL). After incubation at 37 °C for 18 h, the MIC for vancomycin was established as the lowest antibiotic concentration inhibiting bacterial growth and interpreted according to MIC breakpoints established for CoNS by EUCAST. *S. aureus* ATCC25923 was used as the quality control.

### 2.7. Antibiotic Resistance and Virulence Factor Genes

Based on the antibiotic resistance phenotypes, the presence of antimicrobial resistance genes was determined by PCR. In particular, the presence of antimicrobial genes encoding transferable resistance to β-lactam antibiotics (*bla*Z and *mec*A), macrolides and lincosamides (*erm*A, *erm*B, *erm*C, *mph*C, *msr*(A/B), *lnu*A, *lnu*B, and *vga*A), aminoglycosides (*aac(6′)-Ie-aph(2′’)-Ia*, *aph(3′)-*IIIa, *ant(4′)-*Ia, and *str*), fusidic acid (*fus*B), and trimethoprim-sulfamethoxazole (*dfr*A, *dfr*D, *dfr*G, and *dfr*K) was investigated as previously described by Silva et al. *(*2019) [44].

In addition, all isolates were tested by PCR for the presence of virulence genes encoding toxic shock syndrome toxin (*tst*), exfoliative toxins (*eta, etb*, and *etd*2), and the alpha, beta, and delta hemolysins (*hla*, *hlb*, and *hld*, respectively). Moreover, the *scn* gene, which is the marker of the immune evasion cluster (IEC) system, was also investigated. When the *scn* gene was detected, the presence of *chp, sak, sea*, and *sep* was checked to determine the IEC group [44,45,46,47,48].

The positive and negative controls used in PCR assays belonged to the collection of bacterial strains from the Microbiology and Antibiotic Resistance Team (MicroART) of the University of Trás-os-Montes and Alto Douro [49].

### 2.8. Statistical Analyses

Data curation and statistical analyses were performed and graphical representations were generated using the GraphPad Prism 8 software (GraphPad Software, San Diego, CA, USA) and Microsoft Excel (Microsoft Office 365). Statistical analyses were performed using an unpaired Student’s *t*-test to compare biofilm formation amongst the different CoNS isolates at 24 and 48 h.

## 3. Results

### 3.1. Identification of the CoNS Isolated from Fecal and Intestinal Samples from European Hakes

A total of 27 out of the 66 pre-selected isolates from European hake feces and intestines were taxonomically identified as CoNS, namely, *S. epidermidis* (*n* = 16), *S. saprophyticus* (*n* = 4), *S. hominis* (*n* = 3), *S. pasteuri* (*n* = 2), *S. edaphicus (n* = 1), and *S. capitis* (*n* = 1) (Table 1).

### 3.2. Genetic Diversity Analysis by ERIC-PCR

**Figure 1:**
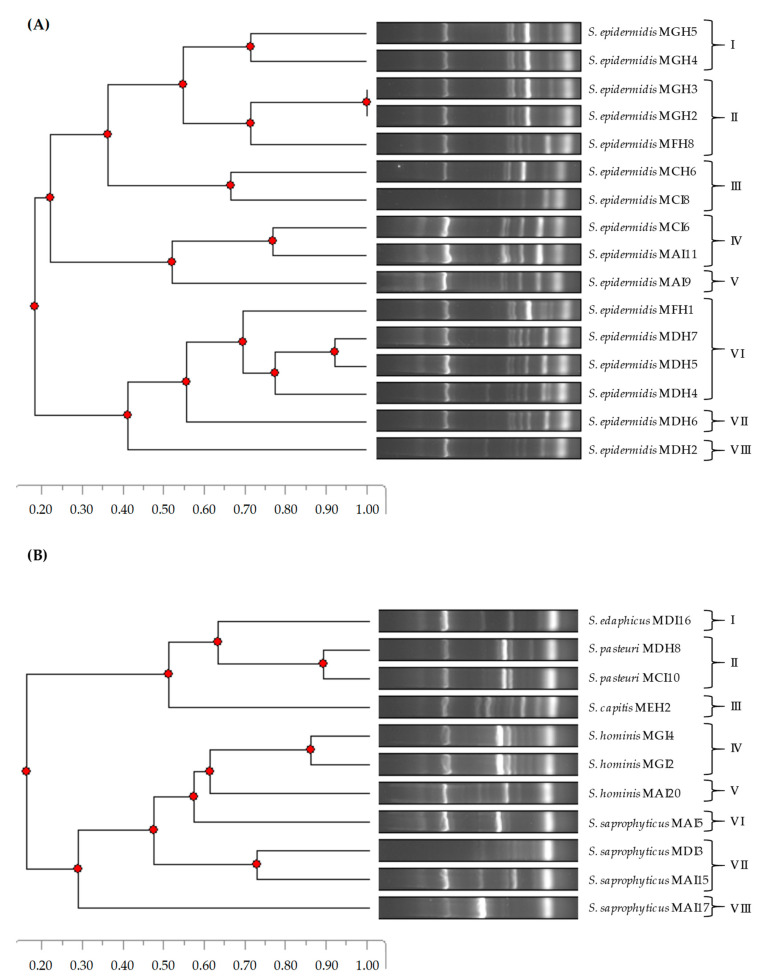
Phylogenetic relatedness of *S. epidermidis* (**A**) and other CoNS species (**B**) isolated from European hakes based on ERIC-PCR patterns.

The phylogenetic relatedness of the CoNS isolates was determined using a DNA fingerprinting method, namely, ERIC-PCR (Figure 1). In the case of *S. epidermidis* isolates, eight ERIC-PCR patterns were detected. While patterns I and II were found in the second year (2022), patterns III–V and VII–VIII were detected in the first year (2021). Interestingly, pattern VI was found in both years. On the other hand, eight ERIC-PCR patterns were identified for the remaining CoNS species. Regarding this, the *S. edaphicus*, *S. pasteuri*, and *S. capitis* isolates were clustered in patterns I, II, and III, respectively. In addition, the *S. saprophyticus* and *S. hominis* isolates were grouped in patterns IV–V and VI–VIII, respectively.

### 3.3. Biofilm Formation

**Figure 2:**
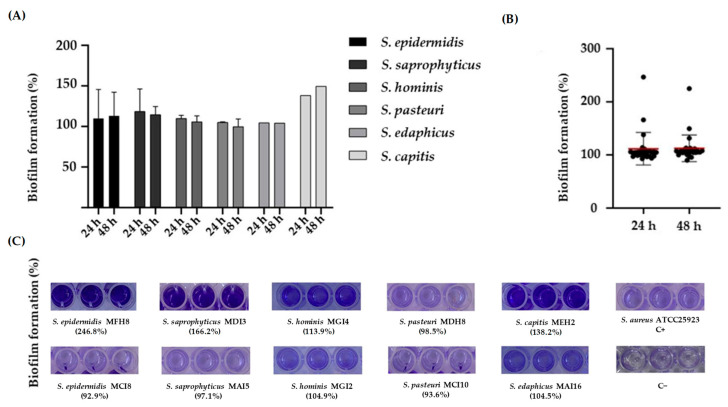
(**A**) Percentage of biofilm formation of the CoNS evaluated in this study at 24 and 48 h. (**B**) Comparison of biofilm formation at 24 and 48 h. The symbols represent the mean of the biofilm formed by the individual isolates. The red lines represent the average of the biofilm mass formed by all the isolates. No significant differences in biofilm formation at 24 and 48 h were observed. (**C**) Representative images of crystal violet staining of biofilms formed by CoNS species at 24 h. When there is more than one isolate of each species, the images show the isolates with the highest and lowest percentages of biofilm formation. C+, positive control. C−, negative control.

The biofilm-forming capability of the CoNS was determined using a microtiter assay [22]. In order to standardize the results, the percentage of biofilm formation for each isolate was normalized using *S. aureus* ATCC25923. Our results showed that all the CoNS produced biofilms (Figure 2A,C), and no statistically significant differences were found at 24 and 48 h (Figure 2B).

### 3.4. Antibiotic Resistance and Virulence Factors

The presence of antibiotic resistance genes in the CoNS in relation to the specific phenotype of resistance as well as the presence of virulence genes is summarized in Table 2. Concerning the antibiotic resistance of CoNS, nine different resistance phenotypes were detected. The results showed that 92.6% of the isolates were resistant to at least one antibiotic. The most frequent resistances were detected to penicillin (88.8%), fusidic acid (40.7%), and erythromycin (37%). Only two isolates were considered as multiresistant (*S. epidermidis* MDH2 and *S. epidermidis* MDH5), as they were resistant to at least three classes of antimicrobial agents. Specifically, *S. epidermidis* MDH2 showed resistance to gentamycin, clindamycin, erythromycin, fusidic acid, cefoxitin, kanamycin, and penicillin, and *S. epidermidis* MDH5 to gentamycin, erythromycin, fusidic acid, kanamycin, penicillin, tobramycin, and trimethoprim-sulfamethoxazole. On the other hand, no phenotypic resistance was detected for ciprofloxacin, linezolid, tetracycline, mupirocin, chloramphenicol, or vancomycin.

According to the antibiotic resistance genotypes, 70.4% of the CoNS harbored at least one antibiotic resistance gene. In this regard, the gene involved in the horizontal transfer of resistance to penicillin (*blaZ*) was identified in 18 isolates (66.7%), 10 harbored macrolide and lincosamide resistance genes (*mphC*, *msr(A/B)*, *lnuA*, or *vgaA*), 4 had the fusidic acid resistance gene (*fusB*), and 3 carried the trimethoprim-sulfamethoxazole resistance gene (*dfrA*). All isolates that had resistance to trimethoprim-sulfamethoxazole also carried the *dfr*A gene. Although the phenotypic resistance to aminoglycosides was identified in three isolates (11.1%), none of them harbored *aac(6′)-Ie-aph(2″)-Ia*, *aph(3′)-IIIa*, *ant(4′)-Ia*, or *str*.

Considering the different species of *Staphylococcus* evaluated in this work, *S. epidermidis* showed the highest number of phenotypic resistances (nine out of the 14 tested antibiotics), specifically to gentamycin, clindamycin, erythromycin, fusidic acid, cefoxitin, kanamycin, penicillin, trimethoprim-sulfamethoxazole, and tobramycin (Table 3). Two of the isolates (*S. epidermidis* MFH1 and MFH8) were susceptible to all the tested antibiotics, and only *S. epidermidis* MFH1 did not harbor any virulence factor gene (Table 2). All *S. saprophyticus* isolates (*n* = 4) displayed resistance to penicillin, half of them showed resistance to fusidic acid, and one isolate was resistant to kanamycin. On the other hand, all *S. hominis* isolates (*n* = 3) were resistant to penicillin, and two isolates were resistant to erythromycin. *S. pasteuri* isolates (*n* = 2) showed phenotypic resistance to penicillin only. *S. edaphicus* (*n* = 1) showed resistance to fusidic acid and penicillin, and *S. capitis* (*n* = 1) was the only species resistant to fusidic acid and not to penicillin (Table 3).

**Figure 3:**
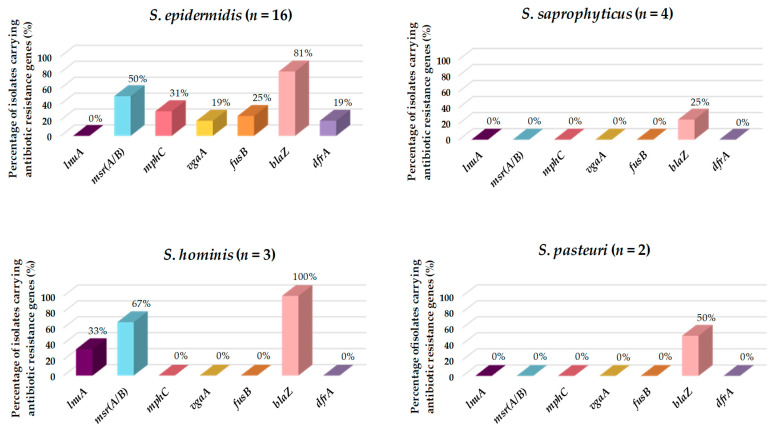
Prevalence (%) of antibiotic resistance genes in the CoNS isolated from European hakes. CoNS species lacking antibiotic resistance genes are not represented.

Of all the CoNS isolated and characterized in this study, *S. epidermidis* was the species harboring the most different resistance genes (*msr(A/B)*, *vgaA*, *fusB*, *blaZ*, *mphC*, and *dfrA*), followed by *S. hominis* (*msr(A/B)*, *blaZ*, and *lnuA*), *S. pasteuri* (*blaZ*), and *S. saprophyticus* (*blaZ*) (Figure 3). However, *S. edaphicus* and *S. capitis* did not harbor any antibiotic resistance genes.

**Figure 4:**
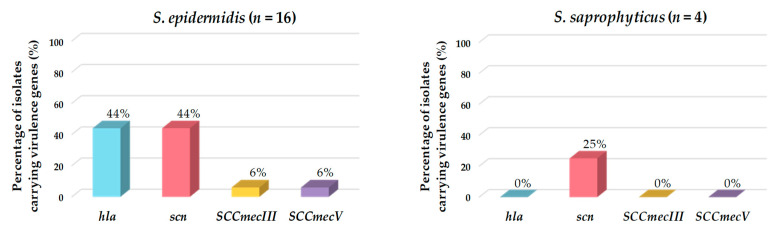
Prevalence (%) of genes encoding virulence factors detected in the CoNS isolated from European hakes. CoNS species lacking virulence genes are not represented.

Of the total CoNS isolates, 48% harbored at least one virulence factor gene. The most frequently detected genes encoding virulence factors were the marker of the IEC system (*scn*) and the hemolysin alpha (*hla*) genes. Of the eight isolates carrying the *scn* gene, seven belonged to the species *S. epidermidis*, and one to *S. saprophyticus*. All isolates carrying *hla*, *SCCmecIII*, or *SCCmecV* were identified as *S. epidermidis* (Table 2 and Figure 4).

## 4. Discussion

Several studies have shown that wastewater treatment does not completely eliminate bacteria, which means that they may reach natural aquatic environments and, subsequently, could be disseminated to animals living in these ecosystems [50,51,52]. As a result, fish and other aquatic animals may act as reservoirs of human pathogenic bacteria of relevance to public health and food safety carrying virulence and antibiotic resistance genes [28,53]. Recent studies have identified antibiotic-resistant and virulent strains of *Staphylococcus* spp. in samples recovered from wastewater treatment plants, supporting the hypothesis that these facilities are one of the main reservoirs of pathogens contributing to their dissemination to aquatic ecosystems [29,51]. Moreover, some studies have described that many cultured fish species are affected by staphylococcal infections worldwide, mostly caused by *S. epidermidis*, *S. aureus*, *S. hominis*, or *S. capitis* [54,55,56]. In this study, CoNS were isolated from fecal and intestinal samples from eight European hake specimens recovered from the Northeast Atlantic, suggesting that the dissemination of staphylococci in the marine environment should not be underestimated. These CoNS isolates were characterized in order to study their capability to form biofilms and to evaluate the presence of genetic determinants conferring antibiotic resistance and virulence.

Molecular fingerprinting methods, such as ERIC-PCR, have widely and successfully been used for bacterial typing and epidemiological studies to determine the genetic relatedness between isolates from various sources, in particular, for *S. aureus* [57], *S. epidermidis*, *S. hominis*, *S. capitis* [58], and other Gram-positive bacteria [39,59]. In this respect, the ERIC-PCR technique represents a valid, fast, and simple strategy for the molecular fingerprinting of isolates. Herein, we show that ERIC-PCR allowed us to differentiate and cluster *S. epidermidis* isolates and to distinguish amongst CoNS species. To the best of our knowledge, this study represents the first description of the use of ERIC-PCR to assess the genetic relatedness in the species *S. edaphicus* and *S. pasteuri.*

Staphylococci, especially CoNS, are generally recognized as the most frequent microorganisms producing biofilm-associated infections [26,60]. All the CoNS isolated from hake feces and intestines showed the ability to form biofilms as previously described for other staphylococci isolated from fish [61,62]. The capability of these microorganisms to produce biofilms in aquatic environment facilitates the exchange of mobile genetic elements amongst aquatic bacteria [50], representing a public health hazard [63,64,65]. Additionally, biofilms facilitate the persistence of pathogenic bacteria in the host and make them resistant to antibiotic treatment [60]. In this respect, *S. epidermidis* is one of the main CoNS species causing nosocomial infections, especially due to its capacity to form biofilms on medical devices in comparison to other biofilm-producing *Staphylococcus* spp. [66]. Similarly, our results showed that *S. epidermidis* harbored the most virulence factor and antibiotic resistance genes. Different studies on the complete genome of this species have concluded that *S. epidermidis* and *S. aureus* share some genes involved in pathogenicity, suggesting the existence of horizontal gene transfer between these species [67]. For this reason, some virulence factors specific to *S. aureus* were evaluated in our study. In this respect, SCC*mec*III and SCC*mec*V were detected in two isolates of *S. epidermidis* (*S. epidermidis* MFH8 and *S. epidermidis* MAI9, respectively). It has been reported that methicillin-resistant staphylococci arise due to the acquisition and insertion of the SCC*mec* element in the chromosome of susceptible strains [68]. Interestingly, only one isolate of this species, *S. epidermidis* MFH1, did not harbor any gene encoding antibiotic resistance or virulence factors.

In addition to *S. epidermidis*, all species studied in this work are potentially pathogenic to both animals and humans. Regarding *S. saprophyticus*, it has been reported that this commensal CoNS has an unusual ability to attach to urothelial cells and produce urease, causing urinary tract infections [69,70,71]. Our results showed that the highest percentages in biofilm-forming isolates were found in this species. However, only one of the four *S. saprophyticus* isolates harbored a virulence factor gene, namely, scn. In this regard, other studies have also described the absence of virulence factors in *S. saprophyticus* [72,73].

*S. capitis* is also a CoNS classified as a human pathogen and involved in infective endocarditis [67], prosthetic joint infections [74], and neonatal sepsis [75]. This species encodes important virulence factors required for biofilm formation, persistence, and immune evasion [76,77,78]. Contrary to these studies, the *S. capitis* isolate from European hake characterized in our work did not harbor any virulence factor genes. With respect to *S. hominis*, it has been recognized as a potentially opportunistic pathogen and may cause bloodstream infections, endocarditis, peritonitis, osteomyelitis, bone, and joint infections [76,79,80,81]. However, the pathogenicity mechanisms of this microorganism have not yet been identified [82]. It should be noted that the *S. hominis* isolates characterized in our study lacked virulence factor genes.

In the case of *S. pasteuri*, it is commonly found in food as well as in the air and on surfaces [83]. Clinically, it has been identified in the gastrointestinal microbiota of children with active celiac disease [84]. In our study, *S. pasteuri* showed resistance to penicillin but did not carry virulence factor genes. Similarly, the only isolate of the species *S. edaphicus* identified in our study showed phenotypic resistance to penicillin and fusidic acid, but no virulence factor genes were found. In this context, Pantůček et al. (2018) described that *S. edaphicus* isolated in Antarctica had penicillin resistance genes, suggesting that this species may act not only as a reservoir of antibiotic resistance in a natural environment but also as a potential source for the spread of antibiotic resistance genes [85].

The uncontrolled use of antibiotics over recent years has led to the emergence of multiresistant *Staphylococcus* spp. strains due to mutations in genes encoding target proteins and, more importantly, through the acquisition and accumulation of genes conferring antibiotic resistance [32]. Studies indicating that food chains are pathways for the transmission of antimicrobial resistance from animals to humans have shown that a high abundance of antibiotic-resistant bacteria and antibiotic resistance genes have been detected in food of animal origin, including fish [86,87,88]. In this study, 92.6% of the CoNS isolates were resistant to at least one antibiotic, and only two multiresistant isolates were identified (*S. epidermidis* MDH2 and *S. epidermidis* MDH5). *Staphylococcus* spp. are frequently resistant to penicillin, followed by fusidic acid [89,90]. This is consistent with our results, in which the most frequent resistances were to penicillin (88.8%) and fusidic acid (40.7%). This can be explained by the data provided in the 2017 ECDC/EFSA/EMA report, in which it was stated that penicillin is one of the most prescribed antibiotics for food-producing animals [91]. *Staphylococcus* spp. exhibit different mechanisms of resistance to β-lactams, such as modified penicillin-binding proteins, production of β-lactamase enzymes, and tolerance phenomena [92]. The most important mechanism is the production of a β-lactamase enzyme, encoded by *blaZ* and controlled by the BlaZ-BlaR1-BlaI system. The genes *blaZ* and those encoding its repressor BlaI and the signal transducer-sensor protein BlaR1 are clustered either on a plasmid or on the bacterial chromosome. In the absence of β-lactam exposure, the DNA repressor BlaI represses *blaZ* expression. However, the detection of β-lactam molecules by BlaR1 initiates a signaling cascade that inhibits the repression of *blaZ* [93,94,95]. In this regard, *blaZ* was detected in 18 out of the 27 CoNS characterized in our work and was the most frequently detected antibiotic resistance gene. In addition, 10 of these isolates harbored macrolide and lincosamide resistance genes (i.e., *mphC*, *msr(A/B)*, *lnuA*, or *vgaA*). The acquisition of resistance to macrolides and lincosamides is mainly due to the following: (i) target-site modification by methylation or mutation that prevents the binding of the antibiotic to its ribosomal target, (ii) efflux of the antibiotic, and (iii) antibiotic inactivation [96]. On the other hand, all the CoNS isolates tested in our study showing trimethoprim-sulfamethoxazole resistance also carried *dfr*A. This is of great interest since this gene is strongly associated with mobile genetic elements such as plasmids and integrons, increasing the dissemination of sulfonamide resistance in aquatic environments [97]. Interestingly, all the CoNS characterized in our study were susceptible to chloramphenicol, ciprofloxacin, linezolid, mupirocin, tetracycline, and vancomycin. Similarly, another study reported that linezolid was effective against CoNS of clinical origin in over 98% of cases [98]. On the contrary, there are recent studies showing concern about resistance to chloramphenicol, ciprofloxacin, and mupirocin in CoNS of clinical origin [99,100,101].

## 5. Conclusions

Our findings show that antibiotic-resistant CoNS harboring virulence factors are present in marine fish, such as European hakes. Specifically, our study reveals a high distribution of biofilm-producing CoNS carrying genes conferring resistance to penicillin (*blaZ*), macrolides and lincosamides (*mphC*, *msr(A/B)*, *lnuA*, or *vgaA*), fusidic acid (*fusB*), and trimethoprim-sulfamethoxazole (*dfrA*) as well as genes encoding virulence traits, namely, *scn*, *hla*, *SCCmecIII*, and *SCCmecV*. Strikingly, only one out of the 27 CoNS isolates (*S. epidermidis* MFH1) was susceptible to all tested antibiotics and lacked virulence factors. Based on our results, the European hakes analyzed in this study, belonging to batches marketed for human consumption, could act as vectors of propagation in the aquatic environment of multiresistant and virulent CoNS potentially pathogenic to animals and humans.

## Figures and Tables

**Table 1:** Number of samples and frequency of the CoNS isolated from European hakes.

Fish	Isolates	Nº CoNS (%)	*S. capitis*	*S. edaphicus*	*S. epidermidis*	*S. hominis*	*S. pasteuri*	*S. saprophyticus*
Hake A	17	7 (41)	0	1	2	1	0	3
Hake B	11	0 (0)	0	0	0	0	0	0
Hake C	4	4 (100)	0	0	3	0	1	0
Hake D	13	7 (53)	0	0	5	0	1	1
Hake E	6	1 (17)	1	0	0	0	0	0
Hake F	3	2 (66)	0	0	2	0	0	0
Hake G	7	6 (86)	0	0	4	2	0	0
Hake H	5	0 (0)	0	0	0	0	0	0
**Total**	66	27 (41)	1	1	16	3	2	4

**Table 2:** Antibiotic resistance and virulence factors of the CoNS isolated from European hakes ^a^.

CoNS	Antibiotic Resistance		Virulence Factors
	Phenotype	Genotype	
*S. saprophyticus* MAI5	FD, PEN	Nf	Nf
*S. epidermidis* MAI9	ERY, FD, PEN	*msr(A/B)*, *vgaA*, *fusB*, *blaZ*	*hla, SCCmecV*
*S. epidermidis* MAI11	ERY, FD, PEN	*mphC*, *msr(A/B)*	*hla*
*S. saprophyticus* MAI15	FD, KAN, PEN	Nf	Nf
*S. edaphicus* MAI16	FD, PEN	Nf	Nf
*S. saprophyticus* MAI17	PEN	Nf	Nf
*S. hominis* MAI20	PEN	*blaZ*	Nf
*S. epidermidis* MCI6	ERY, PEN, SXT	*mphC*, *msr(A/B)*, *blaZ*, *dfrA*	*hla, scn*
*S. epidermidis* MCI8	FD, PEN, TOB	*fusB*, *blaZ*	*hla*
*S. pasteuri* MCI10	PEN	Nf	Nf
*S. epidermidis* MCH6	PEN	*blaZ*	Nf
*S. saprophyticus* MDI3	FD, PEN	*blaZ*	*scn*
*S. epidermidis* MDH2	CN, DA, ERY, FD, FOX, KAN, PEN	*mphC*, *msr(A/B)*, *blaZ*	*hla*
*S. epidermidis* MDH4	ERY, PEN, SXT	*mphC*, *msr(A/B)*, *blaZ*, *dfrA*	Nf
*S. epidermidis* MDH5	CN, ERY, FD, KAN, PEN, TOB, SXT	*mphC*, *msr(A/B)*, *blaZ*, *dfrA*	Nf
*S. epidermidis* MDH6	ERY, FD, PEN	*msr(A/B)*, *vgaA*, *fusB*, *blaZ*	*hla, scn*
*S. epidermidis* MDH7	ERY, FD, PEN	*msr(A/B)*, *vgaA*, *fusB*, *blaZ*	*hla, scn*
*S. pasteuri* MDH8	PEN	*blaZ*	Nf
*S. capitis* MEH2	FD	Nf	Nf
*S. epidermidis* MFH1	Susceptible	Nd	Nf
*S. epidermidis* MFH8	Susceptible	Nd	*SCCmecIII*
*S. hominis* MGI2	ERY, PEN	*msr(A/B)*, *lnuA*, *blaZ*	Nf
*S. hominis* MGI4	ERY, PEN	*msr(A/B)*, *blaZ*	Nf
*S. epidermidis* MGH2	PEN	*blaZ*	*scn*
*S. epidermidis* MGH3	PEN	*blaZ*	*scn*
*S. epidermidis* MGH4	PEN	*blaZ*	*scn*
*S. epidermidis* MGH5	PEN	*blaZ*	*scn*

^a^ Abbreviations: CN, gentamycin; DA, clindamycin; ERY, erythromycin; FD, fusidic acid; FOX, cefoxitin; KAN, kanamycin; PEN, penicillin; SXT, trimethoprim-sulfamethoxazole; TOB, tobramycin; Nf, not found; and Nd, not determined.

**Table 3:** Prevalence (%) of phenotypic antibiotic resistances in the CoNS isolated from European hakes.

Antibiotic ^a^	*S. epidermidis* (*n* = 16)	*S. saprophyticus* (*n* = 4)	*S. hominis* (*n* = 3)	*S. pasteuri* (*n* = 2)	*S. edaphicus* (*n* = 1)	*S. capitis* (*n* = 1)	Total (*n* = 27)
Gentamycin	12.5 (2)	0	0	0	0	0	7.4 (2)
Clindamycin	6.2 (1)	0	0	0	0	0	3.7 (1)
Erythromycin	50 (8)	0	66.6 (2)	0	0	0	37 (10)
Fusidic acid	43.7 (7)	50 (2)	0	0	100 (1)	100 (1)	40.7 (11)
Cefoxitin	6.2 (1)	0	0	0	0	0	3.7 (1)
Kanamycin	12.5 (2)	25 (1)	0	0	0	0	7.4 (2)
Penicillin	87.5 (14)	100 (4)	100 (3)	100 (2)	100 (1)	0	88.8 (24)
Trimethoprim-sulfamethoxazole	18.7 (3)	0	0	0	0	0	11.1 (3)
Tobramycin	12.5 (2)	0	0	0	0	0	7.4 (2)
Sensitive to all antibiotics	12.5 (2)	0	0	0	0	0	7.4 (2)

^a^ All the isolates were sensitive to chloramphenicol, ciprofloxacin, linezolid, mupirocin, tetracycline, and vancomycin.

## Data Availability

Data are available upon request to the corresponding authors.

## Supplementary Materials

The following supporting information can be downloaded at: https://www.mdpi.com/article/10.3390/pathogens12121447/s1; Table S1: Direct antimicrobial activity of the CoNS isolated from European hakes against fish pathogens using the Stab-On-Agar Test (SOAT).
