# Supplementary material for: Antibiotic Resistance Genes, Virulence Factors, and Biofilm Formation in Coagulase-Negative Staphylococcus spp. Isolates from European Hakes (Merluccius merluccius, L.) Caught in the Northeast Atlantic Ocean
Source: Pathogens. 2023 Dec 13;12(12):1447. doi: 10.3390/pathogens12121447 (PMC10745931; doi:10.3390/pathogens12121447)
Supplement: Supplementary file 1 [file pathogens-12-01447-s001.zip › pathogens-2729787-supplementary.pdf]

Table S1. Direct antimicrobial activity of the CoNS isolated from European hakes against fish pathogens using the Stab-On-Agar Test (SOAT)[a].

**CoNS**	***Lc. garvieae* CF00021**	***Lc. garvieae* CLG4**	***L. monocytogenes* CECT911**	***L. ivanovii* CECT913**	***Y. ruckeri* LMG3279**	***A. hydrophila* CECT839**	***A. hydrophila* CECT5734**	***A. salmonicida* CLFP-23**	***A. salmonicida* CECT4237**	***Ls. anguillarum* CECT4344**	***T. maritimum* NCIMB2154**	***T. maritimum* CECT1161**	***E. tarda* CECT886**	***St. parauberis* LMG22225**
S. saprophyticus MAI5	-	++	-	-	-	-		-	-	+	+	-	-	-
S. epidermidis MAI9	+	++	-	-	-	-	-	-	-	+	++	-	-	-
S. epidermidis MAI11	-	++	-	-	-	-	-	-	-	-	-	-	+	+
S. saprophyticus MAI15	+	+	-	-	-	-	-	-	-	-	-	-	-	-
S. edaphicus MAI16	++	+	++	-	-	-	-	-	-	+	-	-	-	-
S. saprophyticus MAI17	-	++	-	-	-	-	-	-	++	+	-	-	-	-
S. hominis MAI20	-	+		-	-	-	-	-	-	+++	++	-	+	+
S. epidermidis MCI6	++	++	+++	++	-	+	+	+	-	-	-	-	+++	+++
S. epidermidis MCI8	-	-	-	-	-	-	-	-	-	-	-	+	+++	+++
S. pasteuri MCI10	-	-	-	++	-	++	-	-	-	-	-	-	+	+
S. epidermidis MCH6	-	-	-	-	-	-	+	+	-	-	-	-	+	+
S. saprophyticus MDI3	-	-	-	-	-	-	-	-	+	+	-	-	-	-
S. epidermidis MDH2	++	++	++	++	-	-	-	-		-	-	+	+++	+++
S. epidermidis MDH4	+	+	++	++	-	-	-	-	+	-	-	+	+++	+++
S. epidermidis MDH5	++	++	++	++	-	-	-	-	-	-	-	+	+++	+++
S. epidermidis MDH6	-	-	-	-	-	-	-	-	-	-	-	-	+	+
S. epidermidis MDH7	-	-	-	-	-	-	-	-	+	-	-	-	-	-
S. pasteuri MDH8	-	-	-	-	-	-	-	-	+	-	-	-	-	-
S. capitis MEH2	-	-	-	-	-	-	-	-	-	-	-	-	++	++
S. epidermidis MFH1	-	-	-	-	-	-	-	+	++	-	-	-	+	+
S. epidermidis MFH8	-	-	-	-	-	-	-	-	-	-	-	-	++	++
S. hominis MGI2	-	-	-	-	-	-	-	-	-	-	-	-	-	-
S. hominis MGI4	-	-	-	-	-	-	-	-	+	-	-	-	-	-
S. epidermidis MGH2	-	-	-	-	+	+	-	-	-	-	-	+	+	+
S. epidermidis MGH3	-	-	-	-	+	+	-	-	+	-	-	+	+	+
S. epidermidis MGH4	-	-	-	-	+	+	-	-	++	-	-	-	+	+
S. epidermidis MGH5	-	-	-	-	+	+	-	-	++	-	-	+	+	+

[a]Scores reflects growth inhibition ranges (inhibition zones diameters in mm): -, no inhibition; +, 3-5 mm; ++, 5-10 mm; +++, 10-15 mm.
